# HIV and syphilis in the context of community vulnerability among indigenous people in the Brazilian Amazon

**DOI:** 10.1186/s12939-017-0589-8

**Published:** 2017-06-05

**Authors:** Adele Schwartz Benzaken, Meritxell Sabidó, Ivo Brito, Ximena Pamela Díaz Bermúdez, Nina Schwartz Benzaken, Enrique Galbán, Rosanna W Peeling, David Mabey

**Affiliations:** 1 Department of STI, AIDS and Viral Hepatitis, Secretary for Health Surveillance, Ministry of Health Brazil, Brasília, DF Brazil; 2 Pan American Health Organization, Brasília, Brazil; 3 TransLab. Department of Medical Sciences, Universitat de Girona, Catalonia, Spain; 4 CIBER of Epidemiology and Public Health (CIBERESP), Madrid, Spain; 5 Departamento de Saúde Coletiva, Universidade de Brasília, Brasília, Brazil; 6 Universidade Nilton Lins, Manaus, Amazonas, Brazil; 7 Facultad de Medicina Calixto García, La Habana, Cuba; 8 Department of Clinical Research, London School of Hygiene and Tropical Medicine, London, UK; 9 Tropical Medicine Foundation Doctor Heitor Vierira Dourado, Manaus, Brazil

**Keywords:** Vulnerability, Syphilis, HIV, Point-of-care testing, Indigenous, Amazon

## Abstract

**Background:**

Contextual factors shape the risk of acquiring human immunodeficiency virus (HIV) and syphilis. We estimated the prevalence of both infections among indigenous people in nine indigenous health districts of the Brazilian Amazon and examined the context of community vulnerability to acquiring these infections.

**Methods:**

We trained 509 health care workers to screen sexually active populations in the community for syphilis and HIV using rapid testing (RT). We then assessed the prevalence of HIV and syphilis using RT. A multivariable analysis was used to identify factors associated with syphilis infection (sociodemographic, condom use, intrusion, population mobility, and violence).

**Results:**

Of the 45,967 indigenous people tested, the mean age was 22.5 years (standard deviation: 9.2), and 56.5% were female. Overall, for HIV, the prevalence was 0.13% (57/43,221), and for syphilis, the prevalence was 1.82% (745/40,934). The prevalence in men, women, and pregnant women for HIV was 0.16%, 0.11%, and 0.07%, respectively, and for syphilis, it was 2.23%, 1.51%, and 1.52%, respectively. The district Vale do Javari had the highest prevalence of both infections (HIV: 3.38%, syphilis: 1.39%). This district also had the highest population mobility and intrusion and the lowest availability of prenatal services. Syphilis infection was independently associated with age (odds ratio [OR] 1.04, 95% confidence interval [CI]: 1.03–1.05), male sex (OR 1.32, 95% CI: 1.14–1.52), and mobility (moderate: OR: 7.46, 95% CI: 2.69–20.67; high: OR 7.09, 95% CI: 3.79–13.26).

**Conclusions:**

The large-scale integration of RT in remote areas increased case detection among pregnant women, especially for syphilis, in districts with higher vulnerability. Mobility is an important risk factor, especially in districts with higher vulnerability. Contextually appropriate approaches that address this factor could contribute to the long-term success of HIV and syphilis control programs.

## Background

Brazil has reported a syphilis prevalence of 0.85% [1] and a human immunodeficiency virus (HIV) prevalence of 0.4% [2]; however, information is scarce on the epidemiology of these infections among indigenous people and regarding which areas or groups should be targeted by prevention efforts [2]. In Brazil, the incidence of congenital syphilis is unacceptable at 4.70 per 1,000 live births, ranking highest in Latin America [1]. The country’s mother-to-child transmission (MTCT) of HIV rate remains high, at 4.4% [1], despite the expansion of HIV testing, with 85% of pregnant women being tested and receiving their results [1].

Vulnerability to STIs among indigenous people can be understood by the intersection of social inequalities, cultural oppression and inter-ethnic friction in a complex mosaic of their sociopolitical environment [3]. At the individual level, knowledge regarding STIs, as well as conceptions, practices and behavior, are key elements to raising awareness and reducing personal vulnerability. This context of vulnerability is influenced by the fact that they are living an epidemiological transition due to their shifting relationship with mainstream society.

Indigenous people are strongly affected by health disparities and social determinants such as limited access to power and resources [4], which may result in a higher prevalence of HIV and syphilis. Community studies in Brazilian Amazonas have revealed a prevalence of syphilis as high as 2.13%; however, HIV prevalence was at 0.13% among indigenous people [5]. Moreover, HIV prevalence in the northern region of the country, where most of the 250 different Brazilian indigenous groups live [6], show increasing trends in the last decade [7]. This increasing HIV prevalence may be due to a wide range of factors, including an adverse socioeconomic context, internal migration flows, inner forms of colonization and expropriation [8], the intensification of large-scale development projects [9], and the opening of new agricultural frontiers in traditional indigenous territories of the Amazon region. All of these factors can be sample characteristics for constructing a vulnerability index [10, 11]. The Amazon region is characterized by most of its population living in small and poorly economically developed towns. Most of the indigenous groups suffer from huge geographic isolation, as these groups are scattered throughout the forest and are accessed only through rivers. Due to these difficulties, STI/HIV prevention interventions for indigenous people is a challenge. Other characteristics, such as poverty, mobility, lack of education and limited access to health services, all of which should be approached from a processes and relational perspective [12], add difficulty to the prevention of STI/HIV. Indigenous women face multiple discriminations on the basis of sex, race/ethnicity, language, culture, religion and class.

This study estimated, for the first time, the prevalence of syphilis and HIV among indigenous people in the Brazilian Amazon and examined the context of community vulnerability to acquiring both infections.

## Methods

### Study setting

This cross sectional study was conducted between February 2009 and June 2011 in nine special indigenous health districts (DSEIs) [13] in the state of Amazonas and Roraima that are accessible only by boat or airplane. Together, these DSEIs serve 227,285 inhabitants and 107 ethnic groups [6].

### Study population and sampling

DSEI coordinators selected a non-random sample of 83 communities. The selection was based on the number of health professionals available to perform testing (at a minimum, nurses and health technicians) and geographic access to the site. The targeted sample size was half of the presumably sexually active subjects based on their age (42,019/84,038). Considering the specific cultural context, the eligible population included potentially sexually active individuals at least 10 years of age, including pregnant women. Those individuals who gave informed consent and accepted rapid test screening were included.

Before the start of the screening activities, health care workers (HCWs) were trained by the staff of the Alfredo da Matta Institute in Manaus. The training covered testing promotion and educational materials, correct handling of testing (finger prick), reading and delivering the results, treatment regimens and patient referrals, and storage conditions. Emphasis was given to confidentiality, respect, and the Brazilian human rights framework on HIV. Support materials to facilitate rapid testing performance were developed.

For quality assurance, HCWs were trained by and provided with proficiency testing panels that included dried tube specimens with sera that were well characterized [14]. The quality control system included standard operating procedures and frequent site-monitoring visits. In each community, a HCW announced the testing through a megaphone as well as by disseminating leaflets and posters that were culturally adapted to each indigenous group. These promotional materials were designed with the involvement of HCWs and indigenous people through an intensive dialog within the different ethnic groups. HCWs provided a health education talk through indigenous community health agents in the native language in place. These agents identified eligible indigenous people living in the community. These communities had no fixed health facility or laboratory, and participants were thus referred to a private area to fulfill confidential testing conditions. Participants signed informed consent, received pre-test HIV counseling by a HCW and were interviewed using a structured questionnaire. Brazilian testing and treatment algorithms for services with no laboratory infrastructure or located in areas difficult to access were followed.

### HIV and syphilis diagnoses

For syphilis diagnosis, the test SD BIOLINE Syphilis 3.0 (Standard Diagnostics, Kyonggi-do, Korea) was used. This rapid treponemal diagnostic tests utilizes immunochromatographic strips and allows onsite screening and treatment [15]. Given that this test has shown both high sensitivity at 0.86 (95% confidence interval [CI] 0.82, 0.89) and high specificity at 0.99 (95% CI 0.94, 1.00), [16] no confirmatory testing was used. For HIV diagnosis, following the Brazilian flow chart, [17] two different rapid tests were used sequentially on different sample finger prick specimens - the DPP HIV-1/2 rapid test from Bio-Manguinhos (Oswaldo Cruz Foundation, Rio de Janeiro, Brazil) and the Rapid Check HIV 1&2 TM (Universidade Federal do Espírito Santo, Vitória, Brazil). If the results between the two tests were discordant, the flow chart was repeated. A HCW collected blood samples using a finger prick, and women received their results within 15 min; they also received free condoms. Nonreactive tests were considered negative. Those with a reactive syphilis rapid test result were immediately treated according to national guidelines, and HIV positive women were referred for CD4 counts and treatment when necessary.

### Data collection

Information on age and condom use during last sexual intercourse (yes/no) with any type of partner was collected by interview using a structured questionnaire. To assess vulnerability to acquiring syphilis in each DSEI, data on levels of intrusion, population mobility, and violence at the DSEI were obtained from secondary sources. Qualitative reports including strategic indicators that covered the study period were used to obtain information about these factors [8, 18, 19]. Based on the combined frequency reported, the presence of these factors was classified as ‘low’, ‘moderate’ or ‘high’. Intrusion was defined as the presence of the timber industry, agro-business, mining activities, or areas of “garimpo”, or informal mining, inside or near the DSEI. Mobility was defined as the frequency of interactions between towns and communities and of contact with travelers, units of the armed forces, and country borders. This definition includes internal mobility that frequently occurs due to rural to urban migration, seasonal and circulatory migration, commuting, and internal displacement. Violence, including sexual violence, was defined as the existence of cases of physical and sexual assault, murders, death threats, other threats, and land disputes. These community level factors were selected because they are considered cross-cutting issues that may influence the way of life of indigenous communities both at the intra- and inter-ethnic level, de-structure their social networks and their internal cohesion, undermine community response [20], and increase susceptibility to HIV and other STIs [21].

### Data analyses

The data were analyzed using STATA version 11.0 (StataCorp, College Station, Texas, USA). The data were described using frequencies, means, and standard deviations (SD) and were presented by DSEI. Each vulnerability factor was classified according to its presence as high, moderate, or low.

A bivariate analysis compared participants infected and non-infected with syphilis on baseline characteristics using the *x*2 test, and crude odds ratios with 95% confidence intervals were calculated. To identify factors independently related to syphilis infection, all factors with a level of significance less than 0.15 were included in a multivariate logistic regression model. To build the final model, a backward procedure was used, and a variable was retained in the model if the likelihood ratio test *P*-value was less than 0.05.

Ethical approval was obtained from the Ethics Board of Fundação Alfredo da Matta (FUAM) (Manaus, Brazil). Parental informed consent for individuals <18 years of age followed national guidelines. In Brazil, according to law CFM n° 1.865/96, if an HIV test is performed on an individual under 18 years of age is voluntarily and with consent, there is no need for authorization or consent from their parents or the responsible person, provided that the adolescent has the capacity to evaluate his/her problem and take actions accordingly.

## Results

### Sample characteristics

Overall, 509 HCWs in 160 health units were trained. A total of 45,967 indigenous people were tested for HIV and syphilis, accounting for 54.7% of the presumably sexually active population (*n* = 84,038) (Table 1). Participants had a mean age of 22.5 years (SD: 9.2), and 25,970 were female (56.5%). The distribution of the indigenous people tested across DSEIs was as follows: 43.3% were from Alto Solimões, and 15.8% were from Alto Rio Negro. Four DSEI contributed to less than 5% of the sample: Vale do Javari (4.1%), Parintins (1.5%), Médio Solimões (1.2%), and Médio Purus (0.7%).

**Table 1:** Characteristics among indigenous people screened according to DSEI, Brazilian Amazon, 2009–2011

DSEI	# screened/sexually active population (%)	Participants screened n (%)	Female sex n (%)	Age in years (mean, SD)	Condom use among those who reported using condoms n (%)	Intrusion	Mobility	Violence
Manaus	8,055/10,980 (73.4)	8,055 (17.5)	4,613 (57.3)	30.6 (14.8)	4,241/4,886 (86.8)	Low	High	High
Yanomami	3,401/4,317 (78.8)	3,401 (7.4)	2,081 (61.4)	27.9 (13.4)	472/1,074 (43.9)	High	Low	High
Leste Roraima	3,892/4,038 (96.4)	3,892 (8.5)	2,569 (66.2)	29.8 (13.2)	858/1,721 (49.9)	High	High	High
Alto Solimões	19,893/25,322 (78.6)	19,893 (43.3)	10,773 (54.2)	25.2 (11.4)	7,150/9,761 (73.3)	High	High	High
Parintins	687/4,904 (14.0)	687 (1.5)	373 (54.5)	30.9 (13.4)	282/457 (61.7)	Low	High	Moderate
Alto Rio Negro	7.260/19,872 (36.5)	7,260 (15.8)	4,117 (56.8)	30.9 (15.2)	1,458/2,867 (50.9)	High	Moderate	High
Médio Solimões	573/9,092 (6.3)	573 (1.2)	321 (56.2)	29.7 (14.1)	329/526 (62.5)	Low	Moderate	Moderate
Médio Purus	329/2,950 (11.2)	329 (0.7)	155 (47.1)	30.0 (13.2)	227/228 (99.6)	Low	Moderate	Moderate
Vale do Javari	1,877/2,563 (73.2)	1,877 (4.1)	968 (57.1)	31.0 (15.9)	1,098/1,329 (82.6)	High	High	High
Total	45,967/84,038 (54.7)	45,967 (100%)	25,970 (56.5)	22.5 (9.2)	16,115/22,849 (70.5)	NA	NA	NA

*NA* not applicable

### HIV and syphilis prevalence

Overall, 745 cases of syphilis were detected among 40,934 indigenous people, resulting in a prevalence of 1.82% (95% CI: 1.69–1.94) (Table 2). By district, the prevalence of syphilis was highest in Vale do Javari (6.14% [95% CI: 5.04–7.23]) (114/1,858), followed by Médio Solimões (3.18% [95% CI: 1.73–4.62]) (18/566) and by Alto Solimões 2.05 (95% CI: 1.85–2.25) (406/19,820). The prevalence in men was 2.23 (95% CI: 2.01–2.44) (394/17,706), in women 1.51 (95% CI: 1.36–1.67) (351/23,208), and in pregnant women 1.52 (95% CI: 1.14–1.89) (63/4,144).

**Table 2:** Syphilis prevalence among indigenous people according to DSEI, Brazilian Amazon, 2009–2011

DSEI	Total sample n/N % (95% CI)	Men n/N % (95% CI)	Women n/N % (95% CI)	Pregnant women n/N % (95% CI)
Manaus	128/7859 1.63 (1.35–1.91)	52/3339 1.56 (1.14–2.00)	76/4515 1.68 (1.31–2.10)	6/460 1.30 (0.27–2.34)
Yanomami	4/2727 0.15 (0.00–0.29)	1/1004 0.10 (0.09–0.29)	3/1722 0.17 (0.42–1.10)	3/703 0.43 (0.06–0.90)
Leste Roraima	33/3777 0.87 (0.57–1.17)	14/1283 1.09 (0.52–1.66)	19/2490 0.76 (0.42–1.10)	4/735 0.54 (0.01–1.10)
Alto Solimões	406/19820 2.05 (1.85–2.25)	233/9082 2.57 (2.24–2.89)	173/10734 1.61 (1.37–1.85)	37/1438 2.57 (1.75–3.39)
Parintins	5/687 0.73 (0.09–0.14)	4/312 1.28 (0.03–2.53)	1/373 0.27 (0.26–0.79)	0/96 0.00 (0.00-0.00)
Alto Rio Negro	37/3319 1.11 (0.76–1.47)	15/1375 1.09 (0.54–1.64)	22/1942 1.13 (0.66–1.60)	7/468 1.50 (0.39–2.60)
Médio Solimões	18/566 3.18 (1.73–4.62)	10/244 4.10 (1.61–6.59)	8/321 2.49 (0.78–4.20)	1/76 1.32 (1.26–3.89)
Médio Purus	0/321 0.0 (0.00-0.00)	0/170 0.00 (0.00-0.00)	0/151 0.00 (0.00-0.00)	0/20 0.00 (0.00-0.00)
Vale do Javari	114/1858 6.14 (5.04–7.23)	65/896 7.25 (5.55–8.93)	49/960 5.10 (3.71–6.50)	5/148 3.38 (0.46–6.30)
Total	745/40934 1.82 (1.69–1.94)	394/17706 2.23 (2.01–2.44)	351/23208 1.51 (1.36–1.67)	63/4144 1.52 (1.14–1.89)

*n/N* number with positive result/number of screened, *%* proportion of positive results among those screened, *CI* confidence interval

The prevalence of HIV was 0.13% (95% CI: 0.10–0.17) (57/43,221), ranging from 0.00% to 0.18% across the DSEIs (Table 3). The prevalence in men was 0.16% (95% CI: 0.10–0.21) (30/18,748), in women 0.11% (95% CI: 0.07–0.15) (27/24,551), and in pregnant women 0.07% (95% CI: 0.00–0.14) (3/4,519). All cases were immediately treated for syphilis or referred for HIV/acquired immune deficiency syndrome (AIDS) treatment as appropriate.

**Table 3:** HIV prevalence among indigenous people according to DSEI, Brazilian Amazon, 2009–2011

DSEI	Total sample n/N % (95% CI)	Men n/N % (95% CI)	Women n/N % (95% CI)	Pregnant women n/N % (95% CI)
Manaus	7/6579 0.11 (0.03–0.19)	6/2756 0.22 (0.04–0.39)	1/3818 0.03 (0.02–0.08)	0/1435 0.00 (0.00-0.00)
Yanomami	6/3256 0.18 (0.14–0.43)	1/1249 0.08 (0.07–0.24)	5/2005 0.25 (0.00–0.47)	0/722 0.00 (0.00-0.00)
Leste Roraima	4/3759 0.11 (0.00–0.21)	0/1260 0.00 (0.00-0.00)	4/2496 0.16 (0.00–0.31)	1/767 0.13 (0.12–0.39)
Alto Solimões	33/19667 0.17 (0.11–0.23)	20/8994 0.22 (0.13–0.32)	13/10669 0.12 (0.06–0.19)	0/1435 0.0 (0.00-0.00)
Parintins	1/685 0.15 (0.14–0.43)	1/311 0.32 (0.31–0.95)	0/372 0.00 (0.00-0.00)	0/96 0.0 (0.00-0.00)
Alto Rio Negro	3/6677 0.04 (0.00–0.10)	2/2894 0.07 (0.03–0.16)	1/3780 0.03 (0.02–0.08)	0/828 0.0 (0.00-0.00)
Médio Solimões	0/572 0.00 (0.00-0.00)	0/250 0.00 (0.00-0.00)	0/321 0.00 (0.00-0.00)	0/76 0.0 (0.00-0.00)
Médio Purus	0/329 0.00 (0.00-0.00)	0/174 0.00 (0.00-0.00)	0/155 0.00 (0.00-0.00)	0/20 0.0 (0.00-0.00)
Vale do Javari	3/1797 0.17 (0.02–0.36)	0/860 0.00 (0.00-0.00)	3/935 0.32 (0.04–0.68)	2/144 1.39 (0.53–3.30)
Total	57/43221 0.13 (0.10–0.17)	30/18748 0.16 (0.10–0.21)	27/24551 0.11 (0.07–0.15)	3/4519 0.07 (0.00–0.14)

*n/N* number with positive result/number of screened, *%* proportion of positive results among those screened, *CI* confidence interval

### Contextual factors

Condom use during last sexual intercourse was reported only by 49.7% of participants (22849/45967). Among those who reported it, condom use during the last sexual intercourse was at 70.5% (Table 1). Among the sub-sample who reported using condoms, in Médio Purus and Manaus, 99.6% and 86.8% of participants reported using a condom during their last sexual act, respectively, but this proportion was lower in the districts of Yanomami (43.9%), Leste Roraima (49.9%), and Alto Rio Negro (50.9%).

Regarding vulnerability, intrusion onto indigenous lands was high in 5 DSEIs (55.6%) and low in 4 DSEIs (44.4%) (Table 1). Population mobility was high in 5 DSEIs (55.6%), moderate in 3 DSEIs (33.4%), and low in 1 DSEI (11.0%). Six DSEIs (66.7%) presented high levels of violence, but in the other 3, levels of violence were moderate (33.3%). Three DSEIs (Leste Roraíma, Alto Solimões, and Vale do Javari) presented high levels of the three vulnerability factors.

### Factors associated with syphilis infection

Tables 4 summarizes the results of the bivariate and multivariate analysis. Variables associated with an increased likelihood of syphilis infection were increasing age, male sex, condom use during last sex, and mobility.

**Table 4:** Factors associated with a positive syphilis result among indigenous people, Brazilian Amazon, 2009–2010: Bivariable and multivariable analysis

	syphilis positive	syphilis negative	*p*-value	Crude OR	*p*-value	Adjusted OR	*p*-value
Age in years (mean, SD)	37.4 (14.1)	27.8 (13.1)	<0.001	1.04 (1.04-1.05)	<0.001	1.05 (1.03–1.05)	<0.001
Sex							
Female	351 (1.5)	22,857 (98.5)	<0.001	1		1	
Male	394 (2.2)	17,312 (97.8)		1.48 (1.28–1.71)	<0.001	1.31 (1.31–1.52)	<0.001
Condom use at last sex							
Yes	349 (2.3)	14,912 (97.7)	<0.001	1			
No	71 (1.2)	5,637 (98.8)		0.54 (0.42–0.70)	<0.001		
Intrusion							
Low	151 (1.6)	9,282 (98.4)		1		1	
Moderate	0 (0.0)	0 (0.0)	0.07				
High	594 (1.9)	30,907 (98.1)		1.18 (0.99–1.41)	0.07	1.80 (1.49–2.18)	<0.001
Mobility							
Low	4 (0.1)	2,723 (99.9)		1		1	
Moderate	55 (1.3)	4,151 (98.7)	<0.001	9.02 (3.27–24.92)	<0.001	7.46 (2.69–20.67)	<0.001
High	686 (2.1)	33,315 (97.9)		14.02 (5.24–37.48)	<0.001	15.95 (5.96–42.71)	<0.001
Violence							
Low	0 (0.0)	0 (0.0)					
Moderate	23 (1.8)	1551 (98.2)	0.28	1			
High	722 (1.8)	38,638 (98.2)		1.26 (0.83–1.91)	0.28		

In the multivariable model, the odds of infection increased by 4% for each additional year of age (OR 1.04, 95% CI: 1.03–1.05, *p* < 0.001). The other factors that remained associated with syphilis infection after adjustment were male sex (OR 1.32, 95% CI: 1.14–1.52, *p* < 0.001) and mobility (moderate: 7.46, 95% CI: 2.69–20.67), and high: OR 7.09, 95% CI: 3.79–13.26, *p* < 0.001).

## Discussion

This study used RT to screen a large sample of indigenous people in remote areas of the Brazilian Amazon and showed an overall prevalence for HIV of 0.13% and for syphilis of 1.82%. Among pregnant women, the prevalence was 0.07% for HIV and 1.52% for syphilis. The district Vale do Javari had the highest prevalence of both infections (HIV: 3.38%, syphilis: 1.39%). Syphilis infection was independently associated with age, male sex and mobility.

These results highlight a high prevalence of syphilis, especially gestational syphilis among indigenous women. The prevalence of 1.52% is twice as high as the national average in Brazilian pregnant women at the time of the study (0.85%) [1]. In the neighboring department of Loreto in the Peruvian Amazonas, indigenous pregnant women had a similar syphilis prevalence of 1.60% and a slightly higher HIV prevalence, 0.16%, than that in our study [22]. The prevalence found is far from the goal of the Elimination Initiative of MTCT of syphilis that aimed to reduce the incidence of congenital syphilis to 0.5 cases or less per 1,000 live births (≤0.05%) by 2015 [1]. In Brazil, the elimination of MTCT of syphilis appeared to have reached a stationary point. The coverage of screening tests for syphilis in pregnant women who attended prenatal care has remained stable at approximately 88% from 2010 to 2014. [1] The percentage of pregnant women with syphilis receiving treatment in Brazil increased slightly from 81% in 2011 to 86% in 2014 [1]. Brazil made progress, although efforts need to continue to eliminate MTCT of syphilis [1]. Despite the high prevalence of syphilis, the prevalence of HIV was low at 0.13%, in contrast with the national HIV prevalence of 0.4% [2]. In indigenous communities in various parts of the world, HIV prevalence is low compared to the rates of syphilis, although STIs are considered strong markers for HIV transmission [23].

Ethnic groups in Vale do Javari were the most affected by syphilis with a substantially higher prevalence of syphilis than in other DSEIs. This DSEI also presented high levels of the three vulnerability factors. In Vale do Javari, other studies have shown that indigenous populations are also affected by a substantially higher prevalence of hepatitis B virus infection [24]. Alto Javari is an extensive area characterized by a combination of isolated indigenous communities, strong illegal exploitation of natural resources and, more recently, intense drug trafficking. Local structures have collapsed as a result and have increased the number of confrontations with indigenous people.

In this study, we found low condom use among indigenous populations in the interior Amazon. In our sample, although condom use during last sexual intercourse was at 70.5%, it was reported by only half of participants and could be overestimated. Indigenous people may perceive the condom not as an HIV/STI prevention method but as a pervasive mechanism for birth control [25]. Studies have shown that the cosmogonies of some indigenous groups in the country discourage condom use, stating that the flow of fluids will bring forces to the body [26]. Condoms have been introduced to indigenous cultural settings since the epidemic was identified, and preventive strategies have also followed cultural adaptation to increase condom acceptability. It would also be beneficial to generate culturally sensitive information, education, and communication (IEC) materials on syphilis prevention to improve communication. Indigenous people should have confidentiality ensured to avoid involuntary disclosure of test results. A study examining the context of the early introduction of RT in the Brazilian Amazon showed that training for HCWs should be based on the following principles: the simplification of technical language; the adaptation of content to the health workers’ levels of knowledge of the indigenous context; the inclusion of case studies or questions and difficulties raised by participants to increase the relevance of the training to the field; and the creation of fact sheets and protocols for syphilis testing, treatment, follow-up and counseling to improve communication between stakeholders [27].

In the current study, mobility arose as a relevant vulnerability factor. Mobility may create a context (separation from family, isolation, greater anonymity, separation from the social norms of their community) in which migrant indigenous peoples may engage in risky sexual practices, such as visiting female sex workers or having multiple sexual partners, as well as alcohol consumption [28]. Understanding how local sexual networks change during migration is crucial to understand the impact of migration on STI/HIV transmission and for designing programs for the prevention of syphilis and HIV. Notably, in this area, actions that individuals take regarding STI/HIV prevention (such as condom use) are intimately linked to the notion of ‘trust’, whereby the perception of one’s risk-taking is more related to the dichotomous construction of people viewed as ‘locals’ versus ‘outsiders’ rather than using an implicit epidemiological construct, such as belonging to ‘low-risk’ or ‘high-risk’ groups [29]. In these isolated communities, there is a general lack of ‘trust’ of outsiders, who belong to the ‘risky’ groups; however, unlicensed behavior is acceptable with ‘locals’ [29]. Sexual health education activities promoting condom use are likely more effective when indigenous people move away from their communities than in promoting condom use within communities.

The health sector plays a major role in preventing HIV and syphilis, and the number of services that offer HIV/AIDS prevention and care in the Amazonas is increasing [2]. However, current services cannot meet the full range of needs and address all barriers to access for indigenous people and in getting them to recognize their own social identity and gender-specific issues [10]. Access in this sense does not only mean entrance to the health system but more complex and comprehensive elements that positively impact health care, such as availability, culturally sensitive health services, accessibility, accommodation, affordability and acceptability [30]. This last dimension is highly sensitive for intercultural health services within indigenous communities operating native meaning categories. Culturally competent providers in different care setting levels remain a great challenge for the national health system. The Sub-System for Indigenous Peoples' Health Care is organized in 34 special sanitary health districts across the country and is guided by a comprehensive and culturally sensitive approach at the local level [13]. Within the district, there is a primary health care net of services known as polo base with a multi-professional team at the community level. Including indigenous health professionals in the community screening is essential to gain proximity to indigenous people. This proximity helps remove the language barrier, given that most ethnic groups continue to communicate primarily in their native language and because few speak Portuguese [31]. Another option to remove barriers for screening is offering home-based testing and counseling, which has proven effective for case detection in the interior of the Amazon [5].

Brazil is close to achieving elimination of HIV MTCT; however, although progress has been made, the country has not eliminated the MTCT of syphilis. Eliminating the MTCT of syphilis largely requires that the efforts of the syphilis control program in the Amazon focus on stability of the availability of RT, increasing the acceptability of RT among indigenous people, [32] closing health staff and antenatal service delivery gaps [33], improving their quality [34], and guaranteeing timely access to treatment [35]. Additionally, strategies that can reach social structure dimensions such as gender roles and sexual behavior among men and women together with cultural identities among different ethnic groups in the Amazon region are warranted. However, the symbolic dimension attached to STI infections in these communities presents challenging and sometimes unknown obstacles for health services structures and providers. Self-perception about risk practices and symptoms for STIs and HIV among these ethnic groups remain to be addressed by health care providers [36].

A limitation of this study was the use of non-random samples, which affects the generalizability of the results. Sample distribution was not balanced between districts, and caution must be taken when establishing comparisons. Presenting vulnerability as one of the three ‘drivers’ of HIV and syphilis may miss several key issues resulting from the complex nature of vulnerability. Other important factors that could influence syphilis infection were not measured nor controlled for in the study, such as the number of partners. The data were collected between February 2009 and June 2011; however, no relevant changes that could conceivably influence the results have since been observed in the Amazonas. One of the major drawbacks of all *Treponema*-specific tests, including the syphilis rapid test that was used in this study, is the persistence of test positivity over time, which limits the utility of such tests to measure prevalence, especially in populations that may have been previously tested and treated. Regarding quality assurance, 268 HCWs were tested using 1607 sample tubes for syphilis and 1608 for HIV using dried tube specimens (DTS). Overall, 150 (9.3%) of the syphilis readings and 111 (6.9%) of the HIV readings were incorrectly interpreted [14]. These results helped identify HCWs in need of retraining to improve their performance.

## Conclusions

As a result of this study, the country of Brazil has changed its policy, adopted RT nationwide, and focused its syphilis control priorities on screening indigenous people. Large-scale integration of RT for HIV and syphilis in remote regions was feasible and has increased access and case detection among indigenous people. This was especially relevant for syphilis in DSEIs with high levels of mobility. Contextually appropriate approaches that address this factor may stand a great chance of long-term success in controlling HIV and syphilis in this region.

## Abbreviations

AIDS: Acquired immune deficiency syndrome
CI: Confidence interval
DSEI: Special indigenous health districts
DTS: Dried tube specimens
FUAM: Fundação alfredo da Matta
HCW: Health care worker
HIV: Human immunodeficiency virus
IEC: Information, education, and communication
MTCT: Mother-to-child transmission
OR: Odds ratio
SD: Standard deviation
SESAI: Special secretariat of indigenous health
